# Molecular Characterization of Multidrug-Resistant *Escherichia coli* from Fecal Samples of Wild Animals

**DOI:** 10.3390/vetsci11100469

**Published:** 2024-10-01

**Authors:** Carolina Sabença, Mario Romero-Rivera, Raquel Barbero-Herranz, Roberto Sargo, Luís Sousa, Filipe Silva, Filipa Lopes, Ana Carolina Abrantes, Madalena Vieira-Pinto, Carmen Torres, Gilberto Igrejas, Rosa del Campo, Patrícia Poeta

**Affiliations:** 1 MicroART-Antibiotic Resistance Team, Department of Veterinary Sciences, University of Trás-os-Montes and Alto Douro, 5000-801 Vila Real, Portugal; anacarolina@utad.pt; 2 Department of Genetics and Biotechnology, University of Trás-os-Montes and Alto Douro, 5000-801 Vila Real, Portugal; gigrejas@utad.pt; 3 Functional Genomics and Proteomics Unit, University of Trás-os-Montes and Alto Douro, 5000-801 Vila Real, Portugal; 4 Associated Laboratory for Green Chemistry, University NOVA of Lisbon, 1099-085 Caparica, Portugal; 5 Department of Microbiology, University Hospital Ramón y Cajal, Instituto Ramón y Cajal de Investigación Sanitaria (IRYCIS), 28034 Madrid, Spain; mario.romero2@ues.edu.sv (M.R.-R.); rabarb01@ucm.es (R.B.-H.); rosacampo@yahoo.com (R.d.C.); 6 CRAS—Center for the Recovery of Wild Animals, Veterinary Hospital, University of Trás-os-Montes and Alto Douro, 5000-801 Vila Real, Portugal; rsargo@utad.pt (R.S.); lsousa16@gmail.com (L.S.); fsilva@utad.pt (F.S.); 7 LxCRAS—Centro de Recuperação de Animais Silvestres de Lisboa, 1500-068 Lisboa, Portugal; ana.f.lopes@cm-lisboa.pt; 8 CECAV—Veterinary and Animal Research Centre, University of Trás-os-Montes and Alto Douro, 5000-801 Vila Real, Portugal; carolina.psca@gmail.com (A.C.A.); mmvpinto@utad.pt (M.V.-P.); 9 Department of Veterinary Sciences, University of Trás-os-Montes and Alto Douro, 5000-801 Vila Real, Portugal; 10 Associate Laboratory for Animal and Veterinary Science (AL4AnimalS), 5000-801 Vila Real, Portugal; 11 Area of Biochemistry and Molecular Biology, OneHealth-UR Research Group, University of La Rioja, 26006 Logroño, Spain; carmen.torres@unirioja.es; 12 Centro de Investigación Biomédica en Red de Enfermedades Infecciosas (CIBERINFEC), Instituto de Salud Carlos III, 28040 Madrid, Spain; 13 Facultad de Ciencias de la Salud, Universidad Alfonso X El Sabio, 28691 Villanueva de la Cañada, Spain

**Keywords:** wildlife, *Escherichia coli*, MDR, ESBL, whole genome sequencing

## Abstract

**Simple Summary:**

The present work primarily focuses on the monitoring of antimicrobial resistance from fecal *Escherichia coli* from wildlife, an important environmental task due to the spread of resistant bacteria. In total, 128 *E. coli* isolates recovered from 66 wild animals were analyzed. Their resistance was tested against 17 antibiotics. An approximate percentage of 22.1% of the animals was found to carry multidrug-resistant *E. coli*, and 0.93% carried strains producing extended-spectrum β-lactamase (ESBL). The highest resistance was observed against ampicillin; all of the isolates were susceptible to amikacin and carbapenems. Therefore, such findings bring concerns about the dissemination of resistant bacteria among wildlife and any further impacts on public health.

**Abstract:**

Antimicrobial resistance (AMR) surveillance in fecal *Escherichia coli* isolates from wildlife is crucial for monitoring the spread of this microorganism in the environment and for developing effective AMR control strategies. Wildlife can act as carriers of AMR bacteria and spread them to other wildlife, domestic animals, and humans; thus, they have public health implications. A total of 128 *Escherichia coli* isolates were obtained from 66 of 217 fecal samples obtained from different wild animals using media without antibiotic supplementation. Antibiograms were performed for 17 antibiotics to determine the phenotypic resistance profile in these isolates. Extended-spectrum β-lactamase (ESBL) production was tested using the double-disc synergy test, and 29 *E. coli* strains were selected for whole genome sequencing. In total, 22.1% of the wild animals tested carried multidrug-resistant *E. coli* isolates, and 0.93% (2/217) of these wild animals carried *E. coli* isolates with ESBL-encoding genes (*bla*_CTX-M-65_, *bla*_CTX-M-55_, and *bla*_EC-1982_). The *E. coli* isolates showed the highest resistance rates to ampicillin and were fully susceptible to amikacin, meropenem, ertapenem, and imipenem. Multiple resistance and virulence genes were detected, as well as different plasmids. The relatively high frequency of multidrug-resistant *E. coli* isolates in wildlife, with some of them being ESBL producers, raises some concern regarding the potential transmission of antibiotic-resistant bacteria among these animals. Gaining insights into antibiotic resistance patterns in wildlife can be vital in shaping conservation initiatives and developing effective strategies for responsible antibiotic use.

## 1. Introduction

Antimicrobial resistance (AMR) is a major concern worldwide as it reduces the efficacy of antibiotics and increases the risk of infections without possible treatment. *Escherichia coli* is a species of bacteria commonly associated with AMR, which is often found colonizing the intestinal tract of wildlife populations. Wildlife can act as a reservoir of resistance genes that can be spread to humans and domestic animals, posing a public health risk [1]. AMR surveillance in *Escherichia coli* from wildlife has become essential under the ecological concept of One Health [2,3].

*E. coli* is a relevant opportunistic pathogen often associated with various infections, including urinary tract infections, sepsis, and pneumonia, among others [4]. The widespread use of antibiotics has led to the selection of AMR *E. coli* isolates, which threaten human and animal health [5].

AMR surveillance in wildlife can also provide valuable information about the presence and spread of AMR bacteria in the environment [6,7]. Wild animals, such as birds and mammals, can acquire AMR bacteria from different contaminated sources and can later contribute to the dissemination of AMR to other animals [8]. The proximity of humans and animals in many rural and urban areas makes monitoring AMR in wildlife populations essential to understand AMR evolution [9].

The presence of extended-spectrum β-lactamases (ESBL) in isolates from wildlife is a matter of significant concern in both the public health and conservation fields. ESBLs are enzymes produced by certain bacteria that confer resistance to a broad range of β-lactam antibiotics, including broad-spectrum cephalosporins [10]. The presence of ESBL-producing isolates in wildlife points to potential environmental contamination with these resistant strains. Fecal shedding from wildlife can introduce ESBLs and other antibiotic-resistant genes into the environment, which may have broader ecological consequences and contribute to the dissemination of resistance genes [10].

Several challenges are associated with AMR surveillance in wildlife, including the need for specialized equipment and expertise and the difficulty of collecting sufficient samples from wildlife populations [11]. In addition, many wildlife populations are difficult to access and sample, making it challenging to obtain representative samples for AMR analysis. Furthermore, the presence of AMR bacteria in wildlife populations may not always indicate the presence of these bacteria in humans or domestic animals, as different populations may have different levels of exposure to antibiotics [12].

Despite these challenges, surveillance in wildlife is an important tool for understanding the spread of AMR bacteria in the environment and developing effective control strategies [13]. In particular, monitoring wildlife populations can provide valuable information about the origin and spread of AMR bacteria and help to identify risk factors [2].

The main objectives of this study were to evaluate the AMR levels of fecal *E. coli* isolates obtained from different wild animals and to characterize them by genomic analysis. Many previous studies in wildlife have been focused on the detection of specific bacteria/AMR phenotypes (for example, ESBL-*E. coli*) using specific media for AMR *E. coli* recovery (antimicrobial-supplemented media). Nevertheless, in this study, we were interested in analyzing the resistance profiles of the *E. coli* isolates obtained from plates not supplemented with antibiotics to obtain information on the AMR levels and genetic characteristics of non-selected *E. coli* isolates of wildlife.

## 2. Materials and Methods

### 2.1. Study Area and Sampling

**Figure 1:**
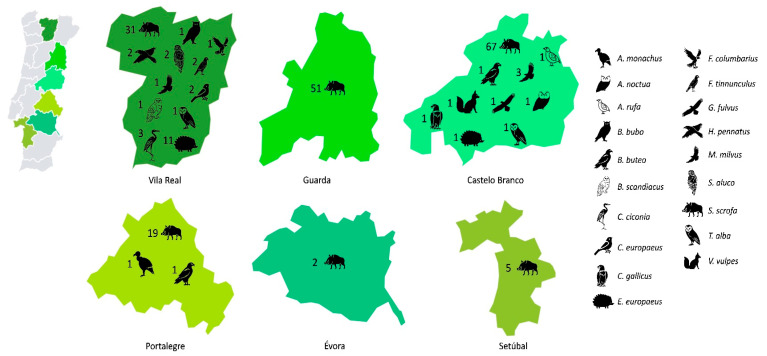
**A map of Portugal showing the area of study (six districts) and the numbers and different species of animals sampled.** The districts are not in scale.

The present study was performed in six different districts of Portugal, namely Vila Real, Guarda, Castelo Branco, Portalegre, Setúbal, and Évora. Fecal samples were collected from 217 different wild animals individually (29 wild birds and 188 mammals) between January 2020 and February 2022 (Figure 1). They were obtained in collaboration with the CRAS (Centro de Recuperação de Animais Selvagens) institution, the CERAS (Centro de Estudos e Recuperação de Animais Selvagens) institution, and during hunts of wild boars organized by different corporations of hunters. Regarding the samples obtained by CRAS and CERAS, the collection of fecal samples was carried out as soon as the animals excreted feces on their own, that is, in the first days of hospitalization, using a spatula and placing the fresh feces directly into a sterilized shipping container. Some of the animals were undergoing antibiotherapy (Supplementary File S1). The wild boar samples were obtained only from those animals which, for the reason of regular hunting, had been legally killed. No animal was killed for the purpose of this study. Fecal samples (~25 g) were collected from the posterior part of the large intestine shortly after death, at evisceration, at the collection point following each driven hunt. They were stored at 4 °C and taken to the laboratory within 12 h.

### 2.2. Bacterial Isolates and Identification

As soon as the samples arrived at the laboratory, 1g of each fecal sample was inoculated in Brain Heart Infusion broth (Liofilchem, Roseto degli Abruzzi, Italy) for enrichment and incubated at 37 °C for 24 h. Then, each sample was streaked on HiCrome *Klebsiella* Selective Agar Base (HiMedia Laboratories, Einhausen, Germany) and incubated for 24 h at 37 °C. In this medium, both *E. coli* and *Klebsiella* spp. grew with the same morphology (purple-magenta color), and no antibiotic was added to specifically select a resistant trait of these species. From each sample, we selected four colonies with this morphology, and we seeded them on Chromogenic Coliform Agar (Oxoid, Cheshire, UK) to differentiate them since *E. coli* grows with a dark blue color and *Klebsiella* spp. develops a salmon-red color. The isolates were identified by MALDI-TOF (Bruker, Billerica, MA, USA), and the *E. coli* isolates were kept for further studies.

### 2.3. Antimicrobial Susceptibility Testing

The phenotypic resistance characterization of the *E. coli* isolates was performed using the Kirby–Bauer disk diffusion method by following EUCAST standards (2022) [14], except for ceftazidime, cefotaxime, tetracycline, and nalidixic acid, for which CLSI standards (2021) were followed [15]. *Escherichia coli* ATCC^®^ 25922 was used as a control strain. The double-disc synergy test was used to evaluate ESBL production. A total of 17 antibiotics were used in the susceptibility testing of *E. coli* isolates (in μg/disk): amoxicillin–clavulanic acid (20–10), amikacin (30), ampicillin (10), aztreonam (30), cefepime (30), cefotaxime (5), cefoxitin (30), ceftazidime (10), chloramphenicol (30), ciprofloxacin (5), ertapenem (10), gentamicin (10), imipenem (10), meropenem (10), nalidixic acid (30), tetracycline (30), and trimethoprim–sulfamethoxazole (1.25/23.75). Mueller–Hinton Agar (Alliance Bio Expertise, Bruz, France) was the medium used for susceptibility testing, and the zones of inhibition were examined after 18 h at 37 °C. Non-repetitive *E. coli* isolates were selected for further studies (only one isolate was selected or more than one if they exhibited different antibiotic resistance profiles). Following this criterion, a collection of 128 non-repetitive *E. coli* isolates were characterized in this study. Isolates that were resistant to three or more antibiotic classes were considered multidrug-resistant (MDR).

### 2.4. Whole Genome Sequencing and Analysis

For whole genome analysis, the main selection criterion for the isolates was their capacity to produce ESBLs. There was a specific interest in the genomic background of antimicrobial resistance, particularly within ESBL-producing isolates, and this was an essential factor that influenced the decision. After that, we further evaluated the total AMR profiles. We prioritized isolates with a wider resistance pattern to have a representative sample of the resistance mechanisms in the population. Finally, the animal species from which the isolates were obtained were included. We aimed for our subset to mimic the variety of animal species and sources available in the populations, with as many isolates from different sources and species included as possible. The whole genome analysis from paired raw reads was realized on selected strains by TORMES v1.3. 0, a bioinformatics pipeline that allows for the quality filtering of raw reads (Prinseq), assembly (SPAdes) and subsequent quality filtering (QUAST), taxonomic identification (Kraken2, RDP Classifier, Barrnap), and annotation (Prokka, Prodigal). In addition, it includes specific programs for typing (MLST, fimHTyper, SerotypeFinder) and searching for resistance genes (Blastn, ABRicate and PointFinder in the databases ResFinder, CARD, ARG-ANNOT and PointFinderDB), virulence factors (ABRicate, VFDB), and plasmid detection (Blastn, ABRicate, PlasmidFinder). Finally, R packages (ggplot2 v3.5.1, ggtree v2.1.0, knitr v1.48, plotly v4.10.4, RColorBrewer v1.1-3, reshape2 v1.4.4, and rmarkdown v2.28) were used to generate the report and the plots that appear in it [16,17,18,19,20,21,22,23,24,25,26,27,28,29,30,31,32,33,34,35,36,37,38,39,40,41,42,43,44]. Phylotyping was conducted using ClermonTyper, which is available online (http://clermontyping.iame-research.center/) (accessed on 27 July 2024).

## 3. Results

### 3.1. E. coli Prevalence in Feces of Wild Animals

A total of 128 non-repetitive *E. coli* isolates were recovered from 66 of the 217 fecal samples collected (30.7%) using media not supplemented with antibiotics. The 66 animals in which *E. coli* isolates could be recovered were the following: 32 wild boars (*Sus scrofa*), 12 hedgehogs (*Erinaceus europaeus*), 2 common kestrels (*Falco tinnunculus*), 3 white storks (*Ciconia ciconia*), 3 red kites (*Milvus milvus*), 1 eurasian eagle-owl (*Bubo bubo*), 3 barn owls (*Tyto alba*), 2 booted eagles (*Hieraaetus pennatus*), 3 tawny owls (*Strix aluco*), 1 common buzzard (*Buteo buteo*), 1 red-legged partridge (*Alectoris rufa*), 1 eurasian griffon vulture (*Gyps fulvus*), 1 red fox (*Vulpes vulpes*), 1 cinereous vulture (*Aegypius monachus*), 1 snowy owl (*Bubo scandiacus*), and 1 merlin (*Falco columbarius*). Of the 66 animals in which *E. coli* isolates were recovered, 16 were undergoing antibiotherapy. Overall, *E. coli* isolates were recovered from 45 wild mammals (23.9%) and 21 wild birds (77.8%) (Table 1).

### 3.2. Antimicrobial Resistance Profile

Firstly, we verified that 71.9% of the *E. coli* isolates were MDR and were obtained from 48 of the 217 animals analyzed (22.1%). From the ESBL production test, we detected two *E. coli* isolates that produced ESBL, obtained from a red fox (*Vulpes vulpes*) (VV1) and Eurasian griffon vulture (*Gyps fulvus*) (GF3) (Table 1); these ESBL-*E. coli* isolates represented 1.6% of the total isolates, 0.9% of animals tested, and 3.0% of animals in which *E. coli* could be recovered. However, 36 isolates were recovered from animals undergoing antibiotherapy, including the ESBL isolate VV1, which could influence the resistance rates of these *E. coli* isolates.

**Figure 2:**
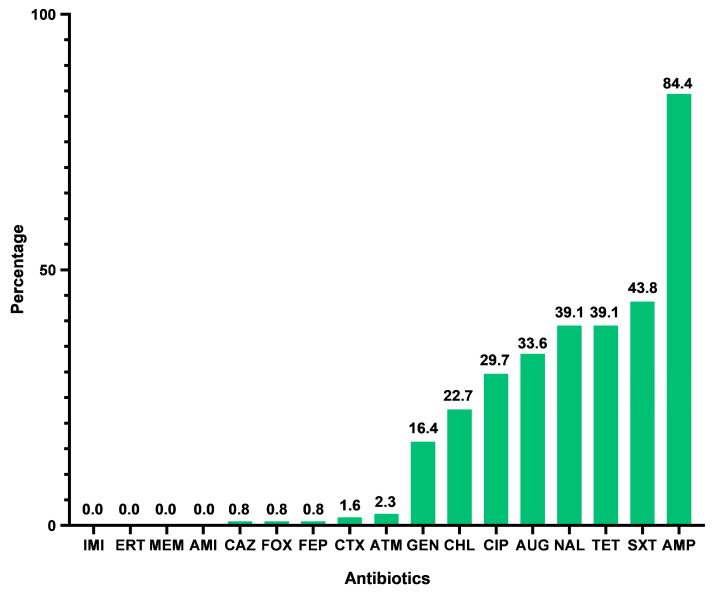
**Percentage of antibiotic resistance in collection of 128 *E. coli* isolates.** IMI—imipenem; ERT—ertapenem; MEM—meropenem; AMI—amikacin; CAZ—ceftazidime; FOX—cefoxitin; FEP—cefepime; ATM—aztreonam; CTX—cefotaxime; GEN—gentamicin; CHL—chloramphenicol; CIP—ciprofloxacin; AUG—amoxicillin–clavulanic acid; NAL—nalidixic acid; TET—tetracycline; SXT—trimethoprim–sulfamethoxazole; AMP—ampicillin.

The *E. coli* isolates, in general, showed high resistance rates to ampicillin (84.4%), trimethoprim–sulfamethoxazole (43.8%), tetracycline (39.1%), nalidixic acid (39.1%), and amoxicillin–clavulanic acid (33.6%); however, they showed low rates of resistance to cefepime (0.8%), cefoxitin (0.8%), and ceftazidime (0.8%) (Figure 2). Among the *E. coli* isolates recovered from the animals undergoing antibiotherapy, we verified that only seven antibiotics (trimethoprim–sulfamethoxazole, nalidixic acid, amoxicillin–clavulanic acid, ciprofloxacin, gentamicin, cefotaxime, and cefoxitin) had higher resistance rates than the *E. coli* isolates recovered from the untreated animals. Additionally, the *E. coli* isolates obtained from treated animals showed the highest resistance rates to ampicillin (91.7%), nalidixic acid (77.8%), amoxicillin–clavulanic acid (63.9%), ciprofloxacin (63.9%), and trimethoprim–sulfamethoxazole (55.6%). The *E. coli* isolates obtained from untreated animals showed the highest resistance rates to ampicillin (100%), tetracycline (54.8%), nalidixic acid (52.4%), trimethoprim–sulfamethoxazole (47.6%), and amoxicillin–clavulanic acid (45.2%).

The isolates exhibited resistance to seven different classes of antibiotics (β-lactams, aminoglycosides, tetracyclines, sulfonamides, trimethoprim–sulfamethoxazole, chloramphenicol, and fluoroquinolones). It was also found that the *E. coli* isolates were resistant to a greater number of antibiotics, such as amoxicillin–clavulanic acid, ampicillin, cefotaxime, chloramphenicol, ciprofloxacin, gentamicin, nalidixic acid, tetracycline, and trimethoprim–sulfamethoxazole. All isolates presented susceptibility to amikacin, meropenem, ertapenem, and imipenem, which are antibiotics that belong to the aminoglycoside and carbapenem classes. More detailed information is given in Supplementary File S1.

### 3.3. Sequencing, Assembly, and Annotation

To assess the carriage of antibiotic resistance and virulence genes, the genomes of 29 representative isolates from one-third of the animals where *E. coli* was obtained (20/66) were sequenced and analyzed. These isolates were recovered from the following animals: 8 wild mammals (1 Vulpes vulpes, 1 Sus scrofa, and 6 Erinaceus europaeus) and 12 wild birds (1 *Aegypius monachus*, 1 *Strix aluco*, 2 *Hieraaetus pennatus*, 1 *Milvus milvus*, 2 *Falco tinnunculus*, 1 *Bubo bubo*, 1 *Tyto alba*, 1 *Falco columbarius*, 1 *Gyps fulvus*, and 1 *Bubo scandiacus*). The average sequencing depth was 588×; the number of assembled contigs ranged between 167 and 7792, and the genome sizes ranged between 4,858,932 and 9,881,410 nucleotides. The average GC content per sample was 50.65%. The draft whole genome sequences of the 29 *E. coli* isolates were deposited in GenBank under the Bio-Project nº PRJNA1006036, and their accession numbers are SAMN37007325, SAMN37007368, and SAMN42001311.

### 3.4. Whole Genome Typing of E. coli Isolates

Among the 29 strains sequenced, we identified 11 sequence types (ST) by multilocus-sequence typing (MLST) based on the seven-gene Achtman’s scheme and 10 FimH types. MLST assignation was not achieved in two strains since new alleles were found or only a partial match to the known allele was made. Of these, ST162, ST1196, and ST2161 were the most prevalent ST observed with five isolates each. Thirteen different serotypes were also identified, and serotypes O100:H28 and O3:H14 were the most frequently detected among the isolates (Table 2). Regarding phylogroups, 5 strains belonged to phylogroup A (17.2%), 22 to phylogroup B1 (75.9%), 1 to phylogroup F (3.4%), and 1 to phylogroup D (3.4%) (Supplementary File S2).

**Figure 3:**
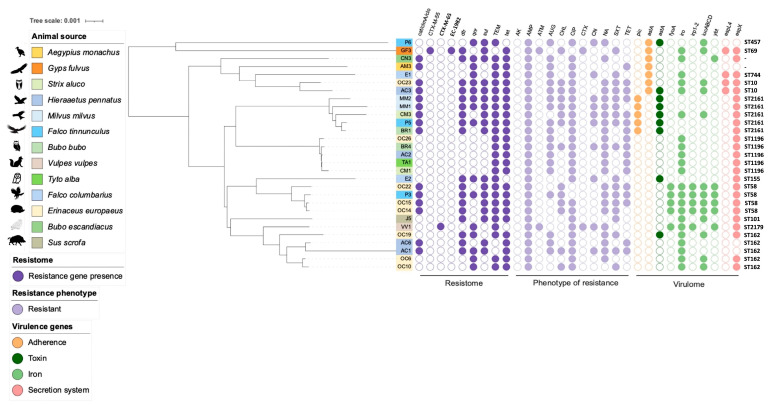
**A pangenome tree based on the core genes’ alignment representation of the genomes of 29 selected *E. coli* isolates.** The presence of resistance genes, resistance phenotypes, and virulence genes is represented by colored circles, and white circles represent their absence. The final column represents the MLST of each isolate. More information on virulence genes is presented in Supplementary File S2.

A pangenome analysis of the 29 *E. coli* genomes generated 2143 core genes, 2810 shell genes, and 13,081 cloud genes. In the core genome cladogram (Figure 3) we can observe the genetic distance between the different isolates. For instance, three *E. coli* isolates (AC1, AC2, and AC3) were obtained from the same wild animal fecal sample [bird *Hieraaetus pennatus* (5077/D1290)]; however, the difference in branch lengths between these isolates suggests significant genetic divergence. Furthermore, AC1 is more genetically related to AC6, an isolate recovered on other animal fecal samples of the same species, than to AC2 and AC3, since they are next to each other on the tree. The same happens between isolates OC22 and OC23; both were isolated from the same fecal sample [mammal *Erinaceus europaeus* (4906/M508)] but are genetically distant. In fact, they are more related to the isolates detected in wild birds (E2, P3, and AC3) than in the other mammals.

### 3.5. Antibiotic Resistance Genes

In agreement with their β-lactam resistance phenotypes, 27 of the 29 selected isolates that were sequenced harbored at least one acquired β-lactamase gene: *bla*_TEM-1B_ (n = 24), *bla*_TEM-1A_ (n = 2), *bla*_CTX-M-65_ (n = 1), *bla*_EC-1982_ (n = 1), and *bla*_CTX-M-55_ (n = 1). The genes encoding CTX-M-65 and CTX-M-55 were detected in two ESBL-positive isolates of the wild animals, namely Vulpes vulpes and Gyps fulvus, respectively (Table 2).

Several other antibiotic resistance genes (ARGs) related to phenicols (*flo*R, *cat*A1, and *cml*A1), aminoglycosides (*aad*A1, *aad*A2, *aad*A5, *aad*A17, *aph*(3′)-Ia, *aph*(3″)-Ib, *aph*(4)-Ia, *aph*(6)-Id, *aac*(3)-Iia, *aac*(3)-Iid, *aac*(3)-Iva, *aac*(3)-IIa, *ant*(3″)-Ia, and *sat*2), tetracyclines (*tet*A and *tet*B), trimethoprim (*dfr*A1, *dfr*A5, *dfr*A12, and *dfr*A17), sulfonamides (*sul*1, *sul*2, and *sul*3), macrolide–lincosamide–streptogramin B (*mph*A, *mph*B, *mph*G, *mef*C, *lnu*F, and *erm*C), quinolones (*qnr*S1, *qnr*S2, *qnr*B5, and *qnr*B19), and the gene responsible for encoding a multidrug transporter (*mdf*A) were found in several isolates. Also, regarding quinolones resistance, different amino acid changes in the GyrA (S83L, D87N, and D87Y), ParC (S80I, A56T, and E84G), and ParE proteins (S458A and L416F) were also found (Table 2).

### 3.6. Plasmid Replicons

At least one plasmid replicon was identified in all isolates, and they corresponded to five incompatibility group plasmids (IncF, IncI, IncR, IncX, and IncN). Col plasmids were identified in 72.4% of the isolates (21/29). The Col440I plasmid was the most frequently detected, and some of the isolates had multiple copies of it. The Col(MG828) (12/29) and ColRNAI (4/29) plasmids were also found. Plasmid IncFIB was frequently identified among the studied isolates (28/29). Most of the isolates (all but one) carried multiple plasmids (Table 2).

### 3.7. Virulence Factors

The 29 sequenced genomes were screened for genes related to virulence factors in the following categories: adherence, iron utilization, secretion systems, and toxins. A total of 22 adherence genes were detected (Supplementary File S2). All strains were positive for the *omp*A gene, a major protein in the *E. coli* outer membrane. Regarding the type 1 fimbriae, the *fim*A/B/C/D/E/F/G/H/I cluster was detected in all but one isolate (E1) where the *fim*A gene was missing from the cluster genes. We also detected *kps*D in the P6 isolate, which is associated with the K1 capsule. All strains harbored the *yag*W/*ecp*D, *yag*X/*ecp*C, *yag*Y/*ecp*B, *yag*Z/*ecp*A, and *ykg*K/*ecp*R genes, which are related to the *E. coli* common pilus. Additional information is available in Supplementary File S2.

A total of 41 iron utilization genes were detected (Supplementary File S2). The *fes* gene that catalyzes the hydrolysis of ferric enterobactin was also identified in all isolates. The *ent*A/B/C/D/E/F/*S* cluster genes were present in 27 strains, and the remaining isolate (P6) lacked the *ent*D gene from this operon. The *fep*A/B/C/D/*G* cluster of genes was present in all isolates. The complete *iro*B/C/D/E/N cluster was detected in 19 strains, but in the AM3 isolate, the *iro*D gene was detected alone, as in the P6 isolate, where the *iro*E gene was also verified independently. One of the twenty-eight studied isolates (P6) harbored the iron uptake cluster genes *chu*S/T/U/V/W/X/Y (missing *chu*A) and also carried the *shu*A gene, an outer membrane receptor that binds to hemoglobin. Additional information is available in Supplementary File S2. Heat-stable enterotoxin 1 (*ast*A) was the only toxin gene present among nine isolates.

A total of 18 secretion system genes were detected. The genes found were involved either in the type II secretion system (T2SS) or the type III secretion system (T3SS). The T2SS widely conserved operon, *gsp*C/D/E/F/G/H/I/J/K/L/M, was present in 26 strains. Among the genes related to T3SS, we detected the *esp*L1/4, *esp*X1/4/5, *esp*R1/4, and *esp*Y1/2/3/4 genes (Supplementary File S2).

## 4. Discussion

The main goal of this study was to evaluate the antimicrobial resistance in *E. coli* from wildlife using a cultivation strategy without antibiotic selection to dimension the actual AMR prevalence among the non-selected *E. coli* isolates from the intestinal tracts of the animals. This information is essential for devising effective strategies to control AMR and for monitoring its progress over time. Given the proximity of humans and animals in various rural and urban settings, monitoring AMR in wildlife populations is crucial to prevent the transmission of MDR bacteria in both directions.

*E. coli* was detected in approximately one-third of the fecal samples analyzed; this was expected as *E. coli* is commonly detected in animals’ intestinal tracts [45,46], and it is more frequently detected in wild bird samples than in mammals. Different rates of *E. coli* recovery were reported in other studies, with higher recovery rates among birds than among mammals in general [8,47].

The relatively frequent detection of MDR or virulent *E. coli* isolates in wild animals is a cause for apprehension since they are able to transmit zoonotic diseases to humans and different animals. Some strains of *E. coli*, such as those with relevant mechanisms of antibiotic resistance (such as ESBL or carbapenemases) or those of virulent serotypes (as O157:H7), can cause zoonotic infections, resulting in various health risks for humans and animals [48].

Only 2 of the 215 tested animals [*Vulpes vulpes* (4492/21) and *Gyps fulvus* (4499/21)] carried *E. coli*-producing ESBL (0.93%). It should be highlighted that we recovered these isolates in a culture medium not supplemented with antibiotics, and consequently, the rate of ESBL carriage in the animals could be underestimated. The low number of wild animals carrying ESBL-positive *E. coli* isolates (n = 2) suggests that the wildlife population may have had limited exposure to these resistant bacteria or their genetic elements carrying ESBL genes, which could indicate that ESBLs are not yet widely spread in the environment where the wildlife was sampled. This result is potentially positive in terms of public health and environmental implications, although surveillance should be continued in the future.

Among the seventeen antibiotics used to test the antibiotic susceptibility of *E. coli* in general, ampicillin had the highest percentage of resistance (84.4%). High resistance rates to ampicillin are frequently detected among *E. coli* strains isolated from wild animals [45,49]. The use of antibiotics in some of the wild animals could have an influence on the percentage of antibiotic resistance since treatment with antibiotics exerts a selective pressure favorable to the survival and proliferation of bacteria possessing mechanisms of resistance to these drugs [50]. The only difference in the highest resistance rates between the treated and untreated animals is that the treated animals were more resistant to ciprofloxacin, and the untreated animals were more resistant to tetracycline. The fact that the treated animals were more resistant to ciprofloxacin is justifiable since one of the antibiotics used to treat these animals is enrofloxacin, which belongs to the same class as ciprofloxacin. Fortunately, most of the research on wild animals has reported, like us, low or no levels of resistance to carbapenem antibiotics [6,45,51,52]. Carbapenems are potent antibiotics often considered a last resort for treating severe bacterial infections. The absence of carbapenem resistance observed in this study in wild animals suggests that this ecosystem has not been contaminated with carbapenem-resistant bacteria, which could be detrimental to wildlife populations.

The genetic relatedness of the *E. coli* isolates in our study was determined by MLST, serotyping, Fim-H typing, and phylotyping of the whole genome sequences of the selected isolates. Our findings show the genetic diversity of the strains as we observed 11 different STs, which is consistent with the results of other studies. However, the most common STs were ST162, ST1196, and ST2161, with each being found in 5 of the 29 selected isolates. ST162 is commonly found among wildlife isolates [53,54], but ST1196 and ST2161 are primarily observed in humans or animals, mainly from poultry samples [55,56,57,58,59]. Still, a survey conducted in Andean condors detected *E. coli* strains belonging to ST1196 [60]. The most frequent serotypes detected were O100:H28 and O3:H14. Due to their presence in multiple reservoirs and their potential to cause infections, serotypes O100 and O3 are frequently found in *E. coli* isolates from animals and humans. The presence of both serotypes in isolates from wild animals highlights the importance of surveillance and control strategies across veterinary and human health sectors. Its ability to spread between animals and humans and the potential for antimicrobial resistance highlights the need for continued monitoring, effective public health interventions, and good education on appropriate food handling practices. Understanding the epidemiological significance of these serotypes is crucial to preventing outbreaks and ensuring public health safety.

The identification of multiple phylogroups within the *E. coli* isolates indicates genetic diversity. The presence of strains belonging to phylogroups A, B1, D, and F aligns with the common phylogroups found in *E. coli*. Commensal strains usually belong to phylogroup A, phylogroup B1 is often found in environmental isolates, and phylogroups D and F may include both commensal and pathogenic strains depending on the specific strain’s virulence factors [61]. The isolate belonging to the phylogroup D was isolated from the species *Gyps fulvus*, which is a species that is exposed to diverse environments potentially harboring and spreading various bacterial strains. This animal was captured by the recovery center of CERAS but was not undergoing antibiotic treatment when the fecal sample was collected, which means it was part of the animal microbiota since no selective pressure was being induced. The circulation of pathogenic *E. coli* strains in the environment poses a zoonotic risk since such strains may infect livestock and humans or be transmitted from wildlife to livestock or human populations, particularly where vultures or other wildlife interact with human populations, farms, or urban environments. Furthermore, the phylogenetic tree showed a great genetic diversity between the 29 *E. coli* isolates, where some isolates detected on mammals were more genetically related to wild bird isolates. This suggests an interesting ecological context since the bacterial strains may be adapted to a specific ecological niche that is conducive to both wild mammals and wild birds. Also, the genetic relatedness may reflect a shared evolutionary history, indicating that these strains have a common ancestry that predates the divergence between these isolates.

The sequence analysis allowed for the detection of a large number of ARGs in the 29 selected *E. coli* isolates. Genes responsible for the production of β-lactamase enzymes were detected, such as *bla*_TEM-1B_, *bla*_TEM-1A_, *bla*_EC-1982_, *bla*_CTX-M-65_, and *bla*_CTX-M-55_. The *bla*_TEM-1B_ and *bla*_TEM-1A_ genes have already been frequently reported in wildlife [62,63,64,65,66,67]. *bla*_EC_ genes encode class C β-lactamase enzymes, which provide resistance to cephalosporins. Many variants of this gene have been identified, but only a few are expressed at levels sufficient to confer antibiotic resistance. [68]. The presence of these variants has been detected in isolates from humans [69] and cattle [70]. The presence of *bla*_CTX-M-55_ and *bla*_CTX-M-65_ in bacteria isolated from wild animals has been documented in several studies [66,71,72,73], which indicates that wild animals may be considered reservoirs for antibiotic-resistant bacteria. Aminoglycoside resistance was confirmed by the presence of several genes that have already been reported among wild animals [74,75,76,77]. However, we detected the *sat*2 gene in one isolate, though it is not usually found in wild animals besides being part of a class 2 integron that carries the classic *dfr*A1, *sat*2, and *aad*A1 gene cassettes. Nonetheless, some studies in wild animals have already reported this gene cassette array [78]. Reports have been frequently made among domestic animals and food products [79,80]. All *E. coli* isolates carried the *mdf*A resistance gene, which confers resistance to a diverse group of cationic compounds and multiple antimicrobial classes, including chloramphenicol, macrolide, lincosamide, and streptogramin, certain aminoglycosides, and fluoroquinolones [81]. The detection of the *mdf*A gene in this study is consistent with other genomic studies characterizing *E. coli* isolates from wildlife [76]. The *tet*A and *tet*B genes, the most widespread and dominant resistant genes detected in enterobacteria across the One Health interface [38,39], were responsible for tetracycline resistance. Other resistance genes that confer resistance to sulfonamides, phenicols, trimethoprim, macrolides, lincosamides, and streptogramin B were also observed. Moreover, we detected chromosomal target mutations in 34 *E. coli* isolates, specifically in the *gyr*A, *par*C, and *par*E genes, which confer resistance to critically important fluoroquinolones [76,82,83]. Besides the mutations responsible for fluoroquinolone resistance, we also detected plasmid-mediated quinolone resistance (PMQR) genes, such as the *qnr*B5, *qnr*B19, *qnr*S1, and *qnr*S2 genes. While *qnr*S1 and *qnr*B19 are relatively more common in wild animals [84], *qnr*S2 [85] and *qnr*B5 [86] are also present but less frequently reported.

The detection of plasmids in all isolates aligns with the well-established understanding that plasmids are common in *E. coli* and other bacteria. Plasmids are extrachromosomal genetic elements that can carry a variety of genes, including those related to antibiotic resistance, virulence, and metabolic functions. The identification of different incompatibility groups (IncF, IncI, IncR, IncX, and IncN) in the isolates is consistent with the diversity of plasmids found in *E. coli* populations [76,87,88], including those of wildlife [3]. Different incompatibility groups represent distinct plasmids, each with its own replication and maintenance mechanisms. Col plasmids can carry genes for the production of colicins, which are bacteriocins that can inhibit the growth of closely related bacteria [89,90]. The detection of Col plasmids in a significant proportion (75.9%) of the isolates is in line with the known presence of colicinogenic plasmids in *E. coli* [76,91]. The identification of the IncFIB plasmid in most isolates is also consistent with the prevalence of IncF-type plasmids in *E. coli* populations [92]. IncF plasmids are known for carrying various genes, including virulence factors and antibiotic resistance genes [93]. In summary, these results regarding the presence of plasmids and their diversity in *E. coli* isolated from wildlife align with the general knowledge in the literature. The variety of incompatibility groups, the presence of common plasmid types, and the occurrence of multiple plasmids in most strains are consistent with the versatility of plasmids in *E. coli* populations.

Regarding the virulence factors, the detection of adherence genes such as *omp*A *and fim*A/B/C/D/E/F/G/H/I align with these genes’ known role in bacterial adherence to host cells and tissues. These genes are associated with forming fimbriae and outer membrane proteins that facilitate adhesion [94,95,96]. The presence of genes related to iron utilization, such as *fes*, *ent*A/B/C/D/E/F/S, and *fep*A/B/C/D/G, is consistent with the need for bacteria to acquire iron from the host environment. These genes are involved in iron acquisition, often through siderophores or heme utilization systems [97]. The detection of the *ast*A gene, which encodes heat-stable enterotoxin 1, in nine isolates is consistent with the presence of this toxin in some strains of *E. coli*. This toxin is associated with gastrointestinal disease [58,98]. The identification of genes related to the type II secretion system (T2SS) and type III secretion system (T3SS) is consistent with the presence of these secretion systems in *E. coli* [99,100]. T2SS is involved in the secretion of various proteins, including toxins, and T3SS is known for delivering effectors into host cells. Overall, these results align with the understanding that different strains of *E. coli* can possess a range of virulence factors related to adherence, iron utilization, toxin production, and secretion systems.

## 5. Conclusions

This study found a relatively high prevalence of MDR *E. coli* isolates among the tested animals (22.3%), corresponding to 71.9% of the total isolates obtained. However, ESBL-positive *E. coli* was only detected in two wild animals. The *E. coli* strains exhibited high rates of resistance to certain antibiotics, specifically ampicillin, trimethoprim–sulfamethoxazole, tetracycline, nalidixic acid, and amoxicillin–clavulanic acid. On the contrary, the resistance rates to cefepime, cefoxitin, and ceftazidime were relatively low. Positively, all *E. coli* strains presented susceptibility to carbapenem antibiotics and also amikacin.

Overall, the results indicate a concerning level of antibiotic resistance in the *E. coli* strains isolated from the wildlife. The detection of MDR and ESBL-producing strains opens the possibility of AMR bacteria transmission among wildlife, other animals, or humans. The finding of full susceptibility to carbapenems is optimistic. Still, continued surveillance and responsible use of antibiotics are essential to prevent the further development and spread of antibiotic resistance in both wildlife and human populations. Additionally, understanding antibiotic resistance patterns in wildlife can help inform conservation efforts and guide strategies for managing antibiotic use in veterinary medicine and agriculture.

Further investigations are necessary to gain a deeper understanding of the antibiotic resistance patterns in wildlife and its potential implications. On the one hand, there is a need to identify the specific mechanisms responsible for antibiotic resistance in the *E. coli* strains isolated from wildlife by understanding the genetic basis of resistance, which can provide insights into how these resistance genes are acquired and spread among bacterial populations. On the other hand, studying the transmission dynamics of antibiotic-resistant *E. coli* between wildlife, humans, and domestic animals is also essential to assess the potential risks of zoonotic transmission. This includes identifying possible reservoirs and vectors of resistant bacteria and understanding the transmission routes. Also, adopting a One Health approach that considers the interconnections between human health, animal health, and the environment is essential to identify the potential sources of antibiotic resistance in wildlife. Investigating the impact of antibiotic resistance on the health and survival of wildlife species is vital for understanding the ecological consequences of resistance development. This can also provide insights into potential risks to biodiversity and ecosystem dynamics. Thus, implementing robust surveillance and monitoring programs is critical to track changes in antibiotic resistance over time in wildlife populations since regularly monitoring resistance patterns can help detect emerging threats and guide effective intervention strategies.

## Figures and Tables

**Table 1:** The wild animal species sampled, the number of *E. coli* isolates obtained, and the number of isolates with an MDR or ESBL phenotype from the respective host species.

Wild Animals				*E. coli* Isolates		
Classes	Species	No. of Animals Sampled	No. of Animals from Which *E. coli* Strains Were Obtained	No.	MDR ^a^	ESBL ^b^
Birds	*Hieraaetus pennatus*	2	2	6	6	0
	*Strix aluco*	3	2	4	4	0
	*Falco columbarius*	1	1	2	2	0
	*Milvus milvus*	4	3	6	6	0
	*Caprimulgus europaeus*	2	0	0	0	0
	*Falco tinnunculus*	2	2	6	5	0
	*Tyto alba*	3	2	5	5	0
	*Bubo bubo*	1	1	2	2	0
	*Ciconia ciconia*	3	3	7	7	0
	*Bubo scandiacus*	1	1	2	1	0
	*Buteo buteo*	2	1	4	3	0
	*Aegypius monachus*	1	1	2	2	0
	*Gyps fulvus*	1	1	2	2	1
	*Athene noctua*	1	0	0	0	0
	*Alectoris rufa*	1	1	2	2	0
	*Circaetus gallicus*	1	0	0	0	0
Mammals	*Erinaceus europaeus*	12	12	27	22	0
	*Vulpes vulpes*	1	1	1	1	1
	*Sus scrofa*	175	32	50	22	0
Total		217	66	128	92	2

^a^ Multidrug Resistant; ^b^ Extended-Spectrum β-Lactamase.

**Table 2:** Comprehensive sequencing data of 29 selected *E. coli* isolates from wild animals.

Strain	Animal of Origin	MLST	Phylogroup	Serotype	FimH Type	Resistome					Plasmids
						β-Lactams	Other Resistance Genes	Quinolone Resistance Due to Point Mutations			
								*gyrA*	*parC*	*parE*	
AC1	*Hieraaetus pennatus* (5077/D1290)	ST162	B1	O25:H9	FimH32	-	*aac*(3)-IIa; *aad*A2; *aad*A5; *ant*(3″)-Ia; *aph*(3″)-Ib; *aph*(3′)-Ia; *mdf*A; *tet*B; *dfr*A17; *sul*2; *cat*A1; *mph*B; *lnu*F; *qnr*B19; *qnr*B5	S83L; D87N	S80I	-	IncFIB; IncFIC(FII); IncI1
AC2		ST1196	B1	O100:H28	FimH31	*bla*_TEM-1B_	*aph*(3″)-Ib; *aph*(6)-Id; *mdf*A; *tet*B; *mph*B	S83L; D87N	S80I	S458A	IncFIB; IncX1
AC3		ST10	A	O101:H10	FimH30	*bla*_TEM-1B_	*aad*A2; *aad*A5; *aad*A1; *mdf*A; *tet*B; *dfr*A17; *sul*1; *sul*3; *cat*A1; *cml*A1; *mph*B; *erm*C; *lnu*F; *qnr*B19; *qnr*B5; *qnr*S1	S83L; D87N	S80I	-	Col(MG828); Col440I (4); IncFIB; IncFIC(FII); IncI1; IncX1; rep10_3_pNE131p1
AC6	*Hieraaetus pennatus* (5121/D1295)	ST162	B1	O25:H9	FimH32	-	*aac*(3)-IIa; *aad*A5; *aad*A2; *ant*(3″)-Ia; *aph*(3″)-Ib; *mdf*A; *tet*B; *dfr*A17; *sul*2; *cat*A1; *mph*B; *lnu*F	S83L; D87N	S80I	-	Col(MG828); Col440I; IncFIB; IncFIC(FII); IncI1
AM3	*Aegypius monachus* (4489/21)	-	A	O101:H4	FimH31	*bla*_TEM-1B_	*aad*A2; *aad*A5; *aph*(3″)-Ib; *aph*(6)-Id; *mdf*A; *tet*B; *cat*A1; *mph*B; *qnr*S1	-	-	-	Col440I; ColRNAI; IncFIB; IncI1; IncR
BR1	*Bubo bubo* (5127/N905)	ST2161	B1	O3:H14	FimH1257	*bla*_TEM-1B_	*aac*(3)-Iva; *aph*(3″)-Ib; *aph*(4)-Ia; *aad*A1; *mdf*A; *tet*A; *dfr*A12; *sul*1; *sul*3; *cml*A1; *mph*B	S83L; D87N	S80I	-	Col(MG828); Col440I (2); IncFIB; IncFIB(K); IncX1
BR4		ST1196	B1	O100:H28	FimH31	*bla*_TEM-1B_	*aph*(3″)-Ib; *aph*(6)-Id; *mdf*A; *tet*B; *mph*B	S83L; D87N	S80I	S458A	Col440I; IncFIB; IncX1
CM1	*Strix aluco* (5119/N903)	ST1196	B1	O100:H28	FimH31	*bla*_TEM-1B_	*aph*(3″)-Ib; *aph*(6)-Id; *mdf*A; *tet*B; *mph*B	S83L; D87N	S80I	S458A	Col440I (2); IncFIB; IncX1
CM3		ST2161	B1	O3:H14	FimH1257	*bla*_TEM-1B_	*aac*(3)-Iva; *aph*(3″)-Ib; *aph*(4)-Ia; *aad*A1; *mdf*A; *tet*A; *dfr*A12; *sul*1; *sul*3; *cml*A1; *mph*B	S83L; D87N	S80I	-	IncFIB; IncFIB(K); IncX1
CN3	*Bubo scandiacus* (5039/N900)	-	A	O25:H9	FimH41	*bla*_TEM-1B_	*aad*A5; *aph*(3″)-Ib; *aph*(6)-Id; *aad*A1; *mdf*A; *tet*A; *dfr*A1; *dfr*A17; *sul*2; *cat*A1; *lnu*F; *mph*B; *qnr*S1	S83L; D87N	S80I; E84G	-	Col440I; IncFIB; IncFIB(pB171); IncI1
E1	*Falco columbarius* (5016/D1215)	ST744	A	O101:H9	FimH54	*bla*_TEM-1B_	*aph*(3″)-Ib; *aph*(3′)-Ia; *aph*(6)-Id; *mdf*A; *tet*B; *mph*B; *qnr*B19; *qnr*B5	S83L; D87N	A56T; S80I	-	IncFIB
E2		ST155	B1	H21	FimH121	*bla*_TEM-1A_	*aac*(3)-IIa; *aad*A1; *mdf*A; *dfr*A1; *sul*1; *mef*C; *mph*B; *mph*G; *qnr*S1	-	-	-	IncI1; IncN
GF3	*Gyps fulvus* (4499/21)	ST69	D	O17/O44:H18	FimH27	*bla*_CTX-M-55_; *bla*_EC-1982_	*aph*(3″)-Ib; *aph*(6)-Id*; mdf*A; *tet*A; *dfr*A5; *sul*2; *flo*R	-	-	-	IncFIB; IncFIC(FII)
MM1	*Milvus milvus* (4923/D1205)	ST2161	B1	O3:H14	FimH1257	*bla*_TEM-1B_	*aac*(3)-Iva; *aad*A2; *aph*(3″)-Ib; *aph*(4)-Ia; *mdf*A; *tet*A; *dfr*A12; *sul*1; *sul*3; *cml*A1; *mph*B; *qnr*B19; *qnr*B5	S83L; D87N	S80I	-	Col(MG828); ColRNAI; IncFIB; IncFIB(K); IncX1
MM2		ST2161	B1	O3:H14	FimH1257	*bla*_TEM-1B_	*aac*(3)-Iva; *aad*A2; *aph*(3″)-Ib; *aph*(4)-Ia; *aad*A1; *mdf*A; *tet*A; *dfr*A12; *sul*1; *sul*3; *cml*A1; *mph*B; *qnr*B19; *qnr*B5	S83L; D87N	S80I	-	Col(MG828); Col440I (3); ColRNAI; IncFIB; IncFIB(K); IncX1
J5	*Sus scrofa* (10P)	ST101	B1	O82:H8	FimH86	*bla*_TEM-1A_	*aad*A1; *sat*2; *mdf*A; *tet*A; *dfr*A1; *sul*3; *mph*B	-	-	-	IncFIA; IncFIB; IncFII; IncI1
OC6	*Erinaceus europaeus* (5049/M521)	ST162	B1	O32:H19	FimH32	*bla*_TEM-1B_	*mph*B; *lnu*F; *mph*B; *qnr*B19; *qnr*B5	S83L; D87N	S80I	-	Col(MG828); Col440I (3); IncFIB; IncFIC(FII)
OC10	*Erinaceus europaeus* (5045/M525)	ST162	B1	O32:H19	FimH32	*bla*_TEM-1B_	*mdf*A; *tet*B; *mph*B; *qnr*B19; *qnr*B5	S83L; D87N	S80I	-	Col440I (2); ColRNAI; IncFIB; IncFIC(FII)
OC14	*Erinaceus europaeus* (4927/M511)	ST58	B1	O9:H25	FimH32	*bla*_TEM-1B_	*aad*A5; *aph*(3″)-Ib; *aph*(6)-Id; *mdf*A; *tet*B; *dfr*A17; *sul*1; *sul*2; *cat*A1; *mph*A; *mph*B	S83L	-	L416F	Col(MG828); Col440I; IncFIB; IncFIC(FII)
OC15		ST58	B1	O9:H25	FimH32	*bla*_TEM-1B_	*aad*A5; *aph*(3″)-Ib; *aph*(6)-Id; *mdf*A; *tet*B; *dfr*A17; *sul*1; *sul*2; *cat*A1; *mph*A; *mph*B	S83L	-	L416F	Col440I; IncFIB; IncFIC(FII)
OC19	*Erinaceus europaeus* (4813/M498)	ST162	B1	O11:H16	FimH32	*bla*_TEM-1B_	*ant*(3″)-Ia; *aad*A2; *mdf*A; *tet*A; *dfr*A17; *lnu*F; *mph*B; *qnr*B19; *qnr*B5	S83L; D87N	S80I	-	Col(MG828); Col440I; IncFIB; IncFIC(FII); IncFII; IncX2
OC22	*Erinaceus europaeus* (4906/M508)	ST58	B1	O9:H25	FimH32	*bla*_TEM-1B_	*aad*A5; *aph*(3″)-Ib; *aph*(6)-Id; *mdf*A; *tet*B; *dfr*A17; *sul*1; *sul*2; *cat*A1; *erm*C; *mph*A; *mph*B	S83L	-	L416F	Col(MG828); Col440I (2); IncFIB; IncFIC(FII); IncI1; rep10_3_pNE131p1
OC23		ST10	A	O101:H10	FimH30	*bla*_TEM-1B_	*aad*A2; *aad*A5; *aad*A1; *mdf*A; *tet*B; *dfr*A17; *sul*1; *sul*3; *cat*A1; *cml*A1; *lnu*F; *mph*B; *qnr*S1	S83L; D87N	S80I	-	Col440I (2); IncFIB; IncFIC(FII); IncI1; IncX1
OC26	*Erinaceus europaeus* (5072/M353)	ST1196	B1	O100:H28	FimH31	*bla*_TEM-1B_	*aph*(3″)-Ib; *aph*(6)-Id; *mdf*A; *tet*B; *mph*B	S83L; D87N	S80I	S458A	Col440I (2); IncFIB; IncX1
P3	*Falco tinnunculus* (4873/D1201)	ST58	B1	O9:H25	FimH32	*bla*_TEM-1B_	*aad*A5; *aph*(3″)-Ib; *aph*(6)-Id; *mdf*A; *tet*B; *dfr*A17; *sul*1; *sul*2; *cat*A1; *mph*A; *mph*B; *qnr*B19; *qnr*B5	S83L	-	L416F	Col440I; IncFIB; IncFIC(FII)
P5	*Falco tinnunculus* (4874/D1202)	ST2161	B1	O3:H14	FimH1257	*bla*_TEM-1B_	*aac*(3)-Iva; *aph*(3″)-Ib; *aph*(4)-Ia; *aad*A1; *mdf*A; *tet*A; *dfr*A12; *sul*1; *sul*3; *cml*A1; *mph*B; *qnr*B19	S83L; D87N	S80I	-	Col(MG828); Col440I (2); IncFIB; IncFIB(K); IncX1
P6		ST457	F	O11:H25	FimH145	*bla*_TEM-1B_	*aac*(3)-IId; *ant*(3″)-Ia; *aad*A17; *mdf*A; *sul*3; *lnu*F; *mef*C; *mph*G; *mph*B; *qnr*B19; *qnr*B5	S83L; D87Y	S80I	S458A	Col(MG828); Col440I (2); IncFIA; IncFIB; IncI1; IncI2; IncX1
TA1	*Tyto alba* (4965/N893)	ST1196	B1	O100:H28	FimH31	*bla*_TEM-1B_	*aph*(3″)-Ib; *aph*(6)-Id; *mdf*A; *tet*B; *mph*B	S83L; D87N	S80I	S458A	Col(MG828); Col440I; IncFIB; IncX1
VV1	*Vulpes vulpes* (4492/21)	ST2179	B1	O9a:H9	FimH32	*bla*_TEM-1B_ *; bla*_CTX-M-65_	*aac*(3)-Iid; *mdf*A; *mph*B; *qnr*S2	S83L	S80I	-	IncFIB; IncFII(29); IncFII(pCoo)

## Data Availability

The datasets generated and/or analyzed in the current study are available in the National Center for Biotechnology Information repository, https://www.ncbi.nlm.nih.gov/bioproject/?term=PRJNA1006036; https://0-www-ncbi-nlm-nih-gov.brum.beds.ac.uk/bioproject/?term=PRJNA1006036 (accessed on 3 September 2024).

## Supplementary Materials

The following supporting information can be downloaded at https://www.mdpi.com/article/10.3390/vetsci11100469/s1, Supplementary File S1: Phenotypic resistance profiles of *E. coli* isolates obtained from wild animals. Supplementary File S2: Virulence genes data of 29 selected *E. coli* isolates from wild animals.

## References

[1] Aslam B., Khurshid M., Arshad M.I., Muzammil S., Rasool M., Yasmeen N., Shah T., Chaudhry T.H., Rasool M.H., Shahid A. (2021). Antibiotic Resistance: One Health One World Outlook. Front. Cell. Infect. Microbiol..

[2] Velazquez-Meza M.E., Galarde-López M., Carrillo-Quiróz B., Alpuche-Aranda C.M. (2022). Antimicrobial resistance: One Health approach. Vet. World.

[3] Alonso C.A., de Toro M., de la Cruz F., Torres C. (2021). Genomic insights into drug resistance and virulence platforms, CRISPR-Cas systems and phylogeny of commensal *E. coli* from wildlife. Microorganisms.

[4] Villafuerte D., Aliberti S., Soni N.J., Faverio P., Marcos P.J., Wunderink R.G., Rodriguez A., Sibila O., Sanz F., Martin-Loeches I. (2020). Prevalence and risk factors for Enterobacteriaceae in patients hospitalized with community-acquired pneumonia. Respirology.

[5] Mancuso G., Midiri A., Gerace E., Biondo C. (2021). Bacterial antibiotic resistance: The most critical pathogens. Pathogens.

[6] Plaza-Rodríguez C., Alt K., Grobbel M., Hammerl J.A., Irrgang A., Szabo I., Stingl K., Schuh E., Wiehle L., Pfefferkorn B. (2021). Wildlife as Sentinels of Antimicrobial Resistance in Germany?. Front. Vet. Sci..

[7] Bengtsson-Palme J., Abramova A., Berendonk T.U., Pedro Coelho L., Forslund S.K., Gschwind R., Heikinheimo A., Hugo Jarquín-Díaz V., Ali Khan A., Klümper U. (2023). Towards monitoring of antimicrobial resistance in the environment: For what reasons, how to implement it, and what are the data needs?. Environ. Int..

[8] Smith S., Wang J., Fanning S., McMahon B.J. (2014). Antimicrobial resistant bacteria in wild mammals and birds: A coincidence or cause for concern?. Ir. Vet. J..

[9] Smoglica C., Vergara A., Angelucci S., Festino A.R., Antonucci A., Marsilio F., Di Francesco C.E. (2023). Antibiotic-Resistant Bacteria Dissemination in the Wildlife, Livestock, and Water of Maiella National Park, Italy. Animals.

[10] Tseng C.-H., Liu C.-W., Liu P.-Y. (2023). Extended-Spectrum β-Lactamases (ESBL) Producing Bacteria in Animals. Antibiotics.

[11] Prestinaci F., Pezzotti P., Pantosti A. (2015). Antimicrobial resistance: A global multifaceted phenomenon. Pathog. Glob. Health.

[12] Argudín M.A., Deplano A., Meghraoui A., Dodémont M., Heinrichs A., Denis O., Nonhoff C., Roisin S. (2017). Bacteria from animals as a pool of antimicrobial resistance genes. Antibiotics.

[13] Arnold K.E., Williams N.J., Bennett M. (2016). “Disperse abroad in the land”: The role of wildlife in the dissemination of antimicrobial resistance. Biol. Lett..

[14] The European Committee on Antimicrobial Susceptibility Testing (2022). Breakpoint Tables for Interpretation of MICs and Zone Diameters. Version 12.0. http://www.eucast.org.

[15] CLSI (2021). Performance Standards for Antimicrobial Susceptibility Testing.

[16] Quijada N.M., Rodríguez-Lázaro D., Eiros J.M., Hernández M. (2019). TORMES: An automated pipeline for whole bacterial genome analysis. Bioinformatics.

[17] Tange O. GNU Parallel 2018. 2018 [Cited 2023 Nov 28]. https://zenodo.org/records/1146014.

[18] Schmieder R., Edwards R. (2011). Quality control and preprocessing of metagenomic datasets. Bioinformatics.

[19] Nurk S., Bankevich A., Antipov D., Gurevich A.A., Korobeynikov A., Lapidus A., Prjibelski A.D., Pyshkin A., Sirotkin A., Sirotkin Y. (2013). Assembling single-cell genomes and mini-metagenomes from chimeric MDA products. J. Comput. Biol..

[20] Gurevich A., Saveliev V., Vyahhi N., Tesler G. (2013). QUAST: Quality assessment tool for genome assemblies. Bioinformatics.

[21] Seemann T. (2018). Barrnap: BAsic Rapid Ribosomal RNA Predictor. https://github.com/tseemann/barrnap.

[22] Wood D.E., Lu J., Langmead B. (2019). Improved metagenomic analysis with Kraken 2. Genome Biol..

[23] Wang Q., Garrity G.M., Tiedje J.M., Cole J.R. (2007). Naïve Bayesian classifier for rapid assignment of rRNA sequences into the new bacterial taxonomy. Appl. Environ. Microbiol..

[24] Seemann T. (2022). mlst: Scan Contig Files Against Traditional PubMLST Typing schemes. https://github.com/tseemann/mlst.

[25] Seemann T. (2020). ABRicate: Mass Screening of Contigs for Antimicrobial Resistance or Virulence Genes. https://github.com/tseemann/abricate.

[26] Zankari E., Hasman H., Cosentino S., Vestergaard M., Rasmussen S., Lund O., Aarestrup F.M., Larsen M.V. (2012). Identification of acquired antimicrobial resistance genes. J. Antimicrob. Chemother..

[27] McArthur A.G., Waglechner N., Nizam F., Yan A., Azad M.A., Baylay A.J., Bhullar K., Canova M.J., De Pascale G., Ejim L. (2013). The Comprehensive Antibiotic Resistance Database. Antimicrob. Agents Chemother..

[28] Gupta S.K., Padmanabhan B.R., Diene S.M., Lopez-Rojas R., Kempf M., Landraud L., Rolain J.M. (2014). ARG-annot, a new bioinformatic tool to discover antibiotic resistance genes in bacterial genomes. Antimicrob. Agents Chemother..

[29] Chen L., Yang J., Yu J., Yao Z., Sun L., Shen Y., Jin Q. (2005). VFDB: A reference database for bacterial virulence factors. Nucleic Acids Res..

[30] Hyatt D., Chen G.L., LoCascio P.F., Land M.L., Larimer F.W., Hauser L.J. (2010). Prodigal: Prokaryotic gene recognition and translation initiation site identification. BMC Bioinform..

[31] Seemann T. (2014). Prokka: Rapid prokaryotic genome annotation. Bioinformatics.

[32] Joensen K.G., Tetzschner A.M.M., Iguchi A., Aarestrup F.M., Scheutz F. (2015). Rapid and easy in silico serotyping of *Escherichia coli* isolates by use of whole-genome sequencing data. J. Clin. Microbiol..

[33] Roer L., Tchesnokova V., Allesoe R., Muradova M., Chattopadhyay S., Ahrenfeldt J., Thomsen M.C.F., Lund O., Hansen F., Hammerum A.M. (2017). Development of a web tool for *Escherichia coli* subtyping based on fimh alleles. J. Clin. Microbiol..

[34] Carattoli A., Zankari E., García-Fernández A., Voldby Larsen M., Lund O., Villa L., Møller Aarestrup F., Hasman H. (2014). In silico detection and typing of plasmids using PlasmidFinder and plasmid multilocus sequence typing. Antimicrob. Agents Chemother..

[35] Zankari E., Allesøe R., Joensen K.G., Cavaco L.M., Lund O., Aarestrup F.M. (2017). PointFinder: A novel web tool for WGS-based detection of antimicrobial resistance associated with chromosomal point mutations in bacterial pathogens. J. Antimicrob. Chemother..

[36] R Core Team (2008). R: A Language and Environment for Statistical Computing.

[37] Wickham H. (2016). ggplot2: Elegant Graphics for Data Analysis.

[38] Yu G., Smith D.K., Zhu H., Guan Y., Lam T.T.Y. (2017). ggtree: An r package for visualization and annotation of phylogenetic trees with their covariates and other associated data. Methods Ecol. Evol..

[39] Xie Y. (2015). Dynamic Documents with R and Knitr.

[40] Sievert C. (2020). Interactive Web-Based Data Visualization with R, Plotly, and Shiny.

[41] Neuwirth E. (2022). RColorBrewer: ColorBrewer Palettes. https://cran.r-project.org/web/packages/RColorBrewer/index.html.

[42] Wickham H. (2007). Reshaping data with the reshape package. J. Stat. Softw..

[43] Allaire J., Xie Y., Dervieux C., McPherson J., Luraschi J., Ushey K., Atkins A., Wickham H., Cheng J., Chang W. (2023). rmarkdown: Dynamic Documents for R. https://github.com/rstudio/rmarkdown.

[44] Wang L.G., Lam T.T.Y., Xu S., Dai Z., Zhou L., Feng T., Guo P., Dunn C.W., Jones B.R., Bradley T. (2020). Treeio: An R Package for Phylogenetic Tree Input and Output with Richly Annotated and Associated Data. Mol. Biol. Evol..

[45] Foti M., Siclari A., Mascetti A., Fisichella V. (2018). Study of the spread of antimicrobial-resistant Enterobacteriaceae from wild mammals in the National Park of Aspromonte (Calabria, Italy). Environ. Toxicol. Pharmacol..

[46] Prandi I., Bellato A., Nebbia P., Stella M.C., Ala U., von Degerfeld M.M., Quaranta G., Robino P. (2023). Antibiotic resistant *Escherichia coli* in wild birds hospitalised in a wildlife rescue centre. Comp. Immunol. Microbiol. Infect. Dis..

[47] Carroll D., Wang J., Fanning S., Mcmahon B.J. (2015). Antimicrobial Resistance in Wildlife: Implications for Public Health. Zoonoses Public Health.

[48] Rahman M.T., Sobur M.A., Islam M.S., Ievy S., Hossain M.J., Zowalaty M.E.E., Rahman A.M.M.T., Ashour H.M. (2020). Zoonotic diseases: Etiology, impact, and control. Microorganisms.

[49] Bamunusinghage N.P.D., Neelawala R.G., Magedara H.P., Ekanayaka N.W., Kalupahana R.S., Silva-Fletcher A., Kottawatta S.A. (2022). Antimicrobial Resistance Patterns of Fecal *Escherichia coli* in Wildlife, Urban Wildlife, and Livestock in the Eastern Region of Sri Lanka, and Differences Between Carnivores, Omnivores, and Herbivores. J. Wildl. Dis..

[50] Larsson D.G.J., Flach C.F. (2022). Antibiotic resistance in the environment. Nat. Rev. Microbiol..

[51] Asai T., Usui M., Sugiyama M., Izumi K., Ikeda T., Andoh M. (2020). Antimicrobial susceptibility of *Escherichia coli* isolates obtained from wild mammals between 2013 and 2017 in Japan. J. Vet. Med. Sci..

[52] Guyomard-Rabenirina S., Reynaud Y., Pot M., Albina E., Couvin D., Ducat C., Gruel G., Ferdinand S., Legreneur P., Le Hello S. (2020). Antimicrobial Resistance in Wildlife in Guadeloupe (French West Indies): Distribution of a Single *bla*CTX–M–1/IncI1/ST3 Plasmid Among Humans and Wild Animals. Front. Microbiol..

[53] Nagy B.J., Balázs B., Benmazouz I., Gyüre P., Kövér L., Kaszab E., Bali K., Lovas-Kiss Á., Damjanova I., Majoros L. (2022). Comparison of Extended-Spectrum Beta-Lactamase-Producing *Escherichia coli* Isolates From Rooks (*Corvus frugilegus*) and Contemporary Human-Derived Strains: A One Health Perspective. Front. Microbiol..

[54] Sellera F.P., Cardoso B., Fuentes-Castillo D., Esposito F., Sano E., Fontana H., Fuga B., Goldberg D.W., Seabra L.A.V., Antonelli M. (2022). Genomic Analysis of a Highly Virulent NDM-1-Producing *Escherichia coli* ST162 Infecting a Pygmy Sperm Whale (*Kogia breviceps*) in South America. Front. Microbiol..

[55] Aworh M.K., Kwaga J., Okolocha E., Harden L., Hull D., Hendriksen R.S., Thakur S. (2020). Extended-spectrum ß-lactamase-producing *Escherichia coli* among humans, chickens and poultry environments in Abuja, Nigeria. One Health Outlook.

[56] Majewski P., Gutowska A., Smith D.G.E., Hauschild T., Majewska P., Hryszko T., Gizycka D., Kedra B., Kochanowicz J., Glowiński J. (2021). Plasmid Mediated *mcr*-1.1 Colistin-Resistance in Clinical Extraintestinal *Escherichia coli* Strains Isolated in Poland. Front. Microbiol..

[57] Aworh M.K., Kwaga J.K.P., Hendriksen R.S., Okolocha E.C., Harrell E., Thakur S. (2023). Quinolone-resistant *Escherichia coli* at the interface between humans, poultry and their shared environment- a potential public health risk. One Health Outlook.

[58] Zhou W., Lin R., Zhou Z., Ma J., Lin H., Zheng X., Wang J., Wu J., Dong Y., Jiang H. (2022). Antimicrobial resistance and genomic characterization of *Escherichia coli* from pigs and chickens in Zhejiang, China. Front. Microbiol..

[59] Grilo T., Freire S., Miguel B., Martins L.N., Menezes M.F., Nordmann P., Poirel L., Sousa M.J.R., Aires-de-Sousa M. (2023). Occurrence of plasmid-mediated fosfomycin resistance (*fos* genes) among *Escherichia coli* isolates, Portugal. J. Glob. Antimicrob. Resist..

[60] Fuentes-Castillo D., Esposito F., Cardoso B., Dalazen G., Moura Q., Fuga B., Fontana H., Cerdeira L., Dropa M., Rottmann J. (2020). Genomic data reveal international lineages of critical priority *Escherichia coli* harbouring wide resistome in Andean condors (*Vultur gryphus* Linnaeus, 1758). Mol. Ecol..

[61] Bhowmik A., Shah S.T., Goswami S., Sirajee A.S., Ahsan S. (2023). Predominance of Multidrug Resistant *Escherichia coli* of Environmental Phylotype in Different Environments of Dhaka, Bangladesh. Trop. Med. Infect. Dis..

[62] Furlan J.P.R., Lopes R., Gonzalez I.H.L., Ramos P.L., Stehling E.G. (2020). Comparative analysis of multidrug resistance plasmids and genetic background of CTX-M-producing *Escherichia coli* recovered from captive wild animals. Appl. Microbiol. Biotechnol..

[63] Martínez-Álvarez S., Châtre P., Cardona-Cabrera T., François P., Sánchez-Cano A., Höfle U., Zarazaga M., Madec J.Y., Haenni M., Torres C. (2023). Detection and genetic characterization of *bla*ESBL-carrying plasmids of cloacal *Escherichia coli* isolates from white stork nestlings (*Ciconia ciconia*) in Spain. J. Glob. Antimicrob. Resist..

[64] Sabença C., Igrejas G., Poeta P., Robin F., Bonnet R., Beyrouthy R. (2021). Multidrug Resistance Dissemination in *Escherichia coli* Isolated from Wild Animals: Bacterial Clones and Plasmid Complicity. Microbiol. Res..

[65] Benavides J.A., Godreuil S., Opazo-Capurro A., Mahamat O.O., Falcon N., Oravcova K., Streicker D.G., Shiva C. (2022). Long-term maintenance of multidrug-resistant *Escherichia coli* carried by vampire bats and shared with livestock in Peru. Sci. Total Environ..

[66] Dalazen G., Fuentes-Castillo D., Pedroso L.G., Fontana H., Sano E., Cardoso B., Esposito F., Moura Q., Matinata B.S., Silveira L.F. (2023). CTX-M-producing *Escherichia coli* ST602 carrying a wide resistome in South American wild birds: Another pandemic clone of One Health concern. One Health.

[67] Zeballos-Gross D., Rojas-Sereno Z., Salgado-Caxito M., Poeta P., Torres C., Benavides J.A. (2021). The Role of Gulls as Reservoirs of Antibiotic Resistance in Aquatic Environments: A Scoping Review. Front. Microbiol..

[68] Schmidt J., Zdarska V., Kolar M., Mlynarcik P. (2023). Analysis of BlaEC family class C beta-lactamase. FEMS Microbiol. Lett..

[69] Tegha G., Ciccone E.J., Krysiak R., Kaphatika J., Chikaonda T., Ndhlovu I., van Duin D., Hoffman I., Juliano J.J., Wang J. (2021). Genomic epidemiology of *Escherichia coli* isolates from a tertiary referral center in lilongwe, Malawi. Microb. Genom..

[70] Adator E.H., Walker M., Narvaez-Bravo C., Zaheer R., Goji N., Cook S.R., Tymensen L., Hannon S.J., Church D., Booker C.W. (2020). Whole genome sequencing differentiates presumptive extended spectrum beta-lactamase producing *Escherichia coli* along segments of the one health continuum. Microorganisms.

[71] Zurfluh K., Albini S., Mattmann P., Kindle P., Nüesch-Inderbinen M., Stephan R., Vogler B.R. (2019). Antimicrobial resistant and extended-spectrum β-lactamase producing *Escherichia coli* in common wild bird species in Switzerland. Microbiologyopen.

[72] Carvalho I., Tejedor-Junco M.T., González-Martín M., Corbera J.A., Suárez-Pérez A., Silva V., Igrejas G., Torres C., Poeta P. (2020). Molecular diversity of Extended-spectrum β-lactamase-producing *Escherichia coli* from vultures in Canary Islands. Environ. Microbiol. Rep..

[73] Zeng Z., Yang J., Gu J., Liu Z., Hu J., Li X., Chen X., Sun Z., Li J. (2022). Prevalence and antimicrobial susceptibility of CTX-M-type-producing *Escherichia coli* from a wildlife zoo in China. Vet. Med. Sci..

[74] Mohsin M., Raza S., Schaufler K., Roschanski N., Sarwar F., Semmler T., Schierack P., Guenther S. (2017). High prevalence of CTX-M-15-Type ESBL-Producing *E. coli* from migratory avian species in Pakistan. Front. Microbiol..

[75] Yang X., Zou W., Zeng J., Xie S., An T., Luo X., Chen D., Feng L., Cheng G., Cai R. (2017). Prevalence of antimicrobial resistance and integron gene cassettes in *Escherichia coli* isolated from yaks (*Poephagus grunniens*) in Aba Tibetan Autonomous Prefecture, China. Microb. Pathog..

[76] Awosile B., Fritzler J., Levent G., Rahman M.K., Ajulo S., Daniel I., Tasnim Y., Sarkar S. (2023). Genomic Characterization of Fecal *Escherichia coli* Isolates with Reduced Susceptibility to Beta-Lactam Antimicrobials from Wild Hogs and Coyotes. Pathogens.

[77] Devnath P., Karah N., Graham J.P., Rose E.S., Asaduzzaman M. (2023). Evidence of Antimicrobial Resistance in Bats and Its Planetary Health Impact for Surveillance of Zoonotic Spillover Events: A Scoping Review. Int. J. Environ. Res. Public Health.

[78] Alonso C.A., Alcalá L., Simón C., Torres C. (2017). Novel sequence types of extended-spectrum and acquired AmpC beta-lactamase producing *Escherichia coli* and *Escherichia* clade V isolated from wild mammals. FEMS Microbiol. Ecol..

[79] Alonso C.A., Michael G.B., Li J., Somalo S., Simón C., Wang Y., Kaspar H., Kadlec K., Torres C., Schwarz S. (2017). Analysis of *blaSHV-12*-carrying *Escherichia coli* clones and plasmids from human, animal and food sources. J. Antimicrob. Chemother..

[80] Hammad A.M., Ishida Y., Shimamoto T. (2009). Prevalence and molecular characterization of ampicillin-resistant Enterobacteriaceae isolated from traditional egyptian domiati cheese. J. Food Prot..

[81] Edgar R., Bibi E. (1997). MdfA, an *Escherichia coli* multidrug resistance protein with an extraordinarily broad spectrum of drug recognition. J. Bacteriol..

[82] Massella E., Reid C.J., Cummins M.L., Anantanawat K., Zingali T., Serraino A., Piva S., Giacometti F., Djordjevic S.P. (2020). Snapshot study of whole genome sequences of *Escherichia coli* from healthy companion animals, livestock, wildlife, humans and food in italy. Antibiotics.

[83] Ong K.H., Khor W.C., Quek J.Y., Low Z.X., Arivalan S., Humaidi M., Chua C., Seow K.L.G., Guo S., Tay M.Y.F. (2020). Occurrence and Antimicrobial Resistance Traits of *Escherichia coli* from Wild Birds and Rodents in Singapore. Int. J. Environ. Res. Public Health.

[84] Skarżyńska M., Zaja̧c M., Bomba A., Bocian Ł., Kozdruń W., Polak M., Wia̧cek J., Wasyl D. (2021). Antimicrobial Resistance Glides in the Sky—Free-Living Birds as a Reservoir of Resistant *Escherichia coli* With Zoonotic Potential. Front. Microbiol..

[85] Literak I., Micudova M., Tausova D., Cizek A., Dolejska M., Papousek I., Prochazka J., Vojtech J., Borleis F., Guardone L. (2012). Plasmid-mediated quinolone resistance genes in fecal bacteria from rooks commonly wintering throughout Europe. Microb. Drug Resist..

[86] Halová D., Papoušek I., Jamborova I., Masarikova M., Cizek A., Janecko N., Oravcova V., Zurek L., Clark A.B., Townsend A. (2014). Plasmid-mediated quinolone resistance genes in enterobacteriaceae from american crows: High prevalence of bacteria with variable *qnrB* genes. Antimicrob. Agents Chemother..

[87] Tarabai H., Krejci S., Karyakin I., Bitar I., Literak I., Dolejska M. (2023). Clinically relevant antibiotic resistance in *Escherichia coli* from black kites in southwestern Siberia: A genetic and phenotypic investigation. mSphere.

[88] Ortega-Balleza J.L., Guerrero A., Castro-Escarpulli G., Martínez-Vázquez A.V., Cruz-Hernández M.A., Luna-Santillana E.d.J.d., Acosta-Cruz E., Rodríguez-Sánchez I.P., Rivera G., Bocanegra-García V. (2023). Genomic Analysis of Multidrug-Resistant *Escherichia coli* Strains Isolated in Tamaulipas, Mexico. Trop. Med. Infect. Dis..

[89] Majeed H., Gillor O., Kerr B., Riley M.A. (2011). Competitive interactions in *Escherichia coli* populations: The role of bacteriocins. ISME J..

[90] Parker J.K., Gu R., Estrera G.A., Kirkpatrick B., Rose D.T., Ai Mavridou D., Mondy K.E., Davies B.W. (2023). Carbapenem-Resistant and ESBL-Producing Enterobacterales Emerging in Central Texas. Infect. Drug Resist..

[91] Moussa J., Nassour E., Tahan E., El Chaar M., Jisr T., Tokajian S. (2023). Carbapenem Resistance Determinants and Their Transmissibility among Clinically Isolated Enterobacterales in Lebanon. J. Infect. Public Health.

[92] Montealegre M.C., Talavera Rodríguez A., Roy S., Hossain M.I., Islam M.A., Lanza V.F., Julian T.R. (2020). High Genomic Diversity and Heterogenous Origins of Pathogenic and Antibiotic-Resistant *Escherichia coli* in Household Settings Represent a Challenge to Reducing Transmission in Low-Income Settings. mSphere.

[93] Stephens C., Arismendi T., Wright M., Hartman A., Gonzalez A., Gill M., Pandori M., Hess D. (2020). F Plasmids Are the Major Carriers of Antibiotic Resistance Genes in Human-Associated Commensal *Escherichia coli*. mSphere.

[94] Connell H., Agace W., Klemm P., Schembri M., Mårild S., Svanborg C. (1996). Type 1 fimbrial expression enhances *Escherichia coli* virulence for the urinary tract. Proc. Natl. Acad. Sci. USA.

[95] Krishnan S., Prasadarao N.V. (2012). Outer membrane protein A and OprF: Versatile roles in Gram-negative bacterial infections. FEBS J..

[96] Day C.J., Lo A.W., Hartley-Tassell L.E., Pilar Argente M., Poole J., King N.P., Tiralongo J., Jennings M.P., Schembri M.A. (2021). Discovery of bacterial fimbria–glycan interactions using whole-cell recombinant *Escherichia coli* expression. MBio.

[97] Mey A.R., Gómez-Garzón C., Payne S.M. (2021). Iron Transport and Metabolism in *Escherichia*, *Shigella*, and *Salmonella*. EcoSal Plus.

[98] Alonso C.A., Mora A., Díaz D., Blanco M., González-Barrio D., Ruiz-Fons F., Simón C., Blanco J., Torres C. (2017). Occurrence and characterization of stx and/or eae-positive *Escherichia coli* isolated from wildlife, including a typical EPEC strain from a wild boar. Vet. Microbiol..

[99] Yang J., Baldi D.L., Tauschek M., Strugnell R.A., Robins-Browne R.M. (2007). Transcriptional regulation of the *yghJ-pppA-yghG-gspCDEFGHIJKLM* cluster, encoding the type II secretion pathway in enterotoxigenic *Escherichia coli*. J. Bacteriol..

[100] Carter M.Q., Quiñones B., Laniohan N., Carychao D., Pham A., He X., Cooley M. (2023). Pathogenicity assessment of Shiga toxin-producing *Escherichia coli* strains isolated from wild birds in a major agricultural region in California. Front. Microbiol..

